# The A2V mutation as a new tool for hindering Aβ aggregation: A neutron and x-ray diffraction study

**DOI:** 10.1038/s41598-017-05582-9

**Published:** 2017-07-14

**Authors:** Laura Cantu’, Laura Colombo, Tatiana Stoilova, Bruno Demé, Hideyo Inouye, Rachel Booth, Valeria Rondelli, Giuseppe Di Fede, Fabrizio Tagliavini, Elena Del Favero, Daniel A. Kirschner, Mario Salmona

**Affiliations:** 1 Department of Medical Biotechnology and Translational Medicine, University of Milan, LITA, Segrate, 20090 Milano, Italy; 2 Department of Molecular Biochemistry and Pharmacology, IRCCS Istituto di Ricerche Farmacologiche “Mario Negri”, Milano, 20156 Italy; 3 Institut Laue-Langevin, Grenoble, Cedex 9, 38042 France; 4 Department of Electrical and Computer Engineering, Northeastern University College of Engineering, Boston, MA 02115 USA; 5 Biology Department, Boston College, Chestnut Hill, MA 02467-3811 USA; 6 Neurology V and Neuropathology Unit, IRCCS Foundation “Carlo Besta” Neurological Institute (INCB), Milano, 20133 Italy

## Abstract

We have described a novel C-to-T mutation in the APP gene that corresponds to an alanine to valine substitution at position 673 in APP (A673V), or position 2 of the amyloid-β (Aβ) sequence. This mutation is associated with the early onset of AD-type dementia in homozygous individuals, whereas it has a protective effect in the heterozygous state. Correspondingly, we observed differences in the aggregation properties of the wild-type and mutated Aβ peptides and their mixture. We have carried out neutron diffraction (ND) and x-ray diffraction (XRD) experiments on magnetically-oriented fibers of Aβ1-28WT and its variant Aβ1-28A2V. The orientation propensity was higher for Aβ1-28A2V suggesting that it promotes the formation of fibrillar assemblies. The diffraction patterns by Aβ1-28WT and Aβ1-28A2V assemblies differed in shape and position of the equatorial reflections, suggesting that the two peptides adopt distinct lateral packing of the diffracting units. The diffraction patterns from a mixture of the two peptides differed from those of the single components, indicating the presence of structural interference during assembly and orientation. The lowest orientation propensity was observed for a mixture of Aβ1-28WT and a short N-terminal fragment, Aβ1-6A2V, which supports a role of Aβ’s N-terminal domain in amyloid fibril formation.

## Introduction

Alzheimer’s disease (AD), the most frequent form of dementia in the elderly, is defined neuropathologically by extracellular amyloid plaques and intraneuronal neurofibrillary tangles in limbic and cortical regions of brain^1^. The principal pathological hallmark of AD is the accumulation of amyloid-β (Aβ) peptides that are misfolded, and assemble as oligomers and fibrils^2–4^. The peptides Aβ1-40 and Aβ1-42 are generated by proteolytic cleavage of a large (695/770 amino acids) type 1 transmembrane glycoprotein known as amyloid β precursor protein (APP)^2, 5^. Aggregated Aβ species are believed to trigger a cascade of events that lead to protein tau hyperphosphorylation, misfolding, and assembly into abnormal filaments of neurofibrillary tangles, disruption of the neuronal cytoskeleton, widespread synaptic loss, and neurodegeneration^6, 7^. This cascade appears to offer a number of points at which to interfere with the sequence of pathology; however, to date all strategies have faced serious hurdles in clinical application, where the tested drugs have been ineffective or caused severe adverse events^8, 9^. Thus, there is still an urgent need for safe and effective molecules for AD treatment.

We have previously identified an APP gene mutation that is consistent with a recessive Mendelian trait of inheritance, causing disease only in the homozygous state and not in heterozygous carriers even when elderly^10^. The mutation consists of a C-to-T transition generating an A-to-V substitution at position 673 of APP, and in position 2 of Aβ. *In vitro* studies with fibroblasts from patients and controls, APP transfected cells and synthetic Aβ peptides show that the A673V APP mutation has two pathogenic effects: (*i*) shifting APP processing toward the amyloidogenic pathway with increased Aβ production, and (*ii*) enhancing the aggregation and fibrillogenic properties of Aβ. However, the interaction between mutant and wild-type Aβ interferes with nucleation or nucleation-dependent Aβ polymerization, or both, hindering amyloidogenesis and Aβ-related neurotoxicity, and thus protecting heterozygous carriers from disease^10, 11^. Correspondingly, we also observed consistent differences in the early-stage aggregation process of the wild-type and mutated Aβ peptides and their mixture^12^. The identification of a mutated Aβ sequence which acts in a dominant-negative fashion on amyloidogenesis has important implications for potential treatment of both the sporadic and familial forms of AD^10, 13^. Distinct from previous approaches based on theoretical grounds, our novel strategy stems from the clinical observation that a naturally-occurring variant of Aβ protects from disease^10, 14, 15^. Notably, in prion protein involved Creutzfeld-Jacob-Disease (CJD), variability has been linked to genetic factors and to the chemico-physical properties of PrP^Sc^. Different PrP^Sc^ “conformers” and distinct polymorphisms at codon 129 site encoding methionine (M) or valine (V), may modulate the susceptibility, the incubation period and the clinical and pathological features of the disease, resulting in the occurrence of different CJD phenotypes^16, 17^.

Preliminary to designing an inhibitor of Aβ aggregation based on the mutated Aβ sequence, we synthesized a short peptide homologous to residues 1–6 of Aβ with and without the A2V substitution and tested their ability to bind preformed Aβ fibrils and to inhibit amyloidogenesis. The mutated but not the wild-type six-mer peptide was found to retain the anti-amyloidogenic properties of the parent full-length Aβ^13^. Molecular dynamics simulations suggest that this property may be related to the structural flexibility of the peptide which adopts dynamically “closed” and “open” configurations, at variance with the wild-type sequence which is characterized by a “closed” structure^11^. The labile structure of the mutated hexapeptide may facilitate heterotypic interaction with Aβ, thereby hindering Aβ assembly. These preliminary experiments offered a proof of concept and provide a foundation for the rational design of therapeutic agents based on Aβ fragments with the A2V mutation and peptido-mimetic molecules retaining the key functional properties of the mutated Aβ. In light of these observations the main questions still to be answered are: (*i*) the structural characterization of macromolecular assemblies of Aβ peptides with the A2V mutation, and (*ii*) the biochemical basis for the aggressiveness of the A2V mutation.

## Results and Discussion

A significant aspect in AD therapeutics is the different response to treatment of the patients in clinical trials. This may be ascribed to amyloid-β fibril polymorphism that is correlated with subgroups of disease neuropathological and biochemical profiles, and pathogenic pathways^18^. Understanding the molecular basis of AD heterogeneity may help design more appropriate therapies based on recognition of different target phenotypes. Therefore, the identification and categorization of AD subtypes is now becoming an urgent need for its potential diagnostic and therapeutic implications.

Here we report on and analyze the diffraction patterns of Aβ1-28 wild type (WT) the mutated peptide Aβ1-28A2V (A2V), their 1:1 molar mixture (WT:A2V) and the mixture of WT with the short mutated C-terminal sequence Aβ1-6A2V (1-6A2V). All peptides were slowly dried on a flat support under a static magnetic field. Morphological characterization by microscopy was performed under different experimental conditions, i.e., on deposited samples and as reconstituted in aqueous solution. Diffraction from WT and A2V in the form of lyophilized powder was also recorded.

The wide-angle x-ray powder diffraction patterns of lyophilized WT and A2V (Supplementary Information, Fig. SI1) showed two spherically-averaged reflections, at spacings of ~10 Å and ~4.7 Å, characteristic of the intersheet and hydrogen-bonding distances, respectively, for a β-sheet conformation. Moreover, like the fibril-forming fragments Aβ1-40 and Aβ1-42 of APP, both the wild type and A2V sequences for the 28 amino acid-long fragment of Aβ1-40/42, assembled as fibrils. Analysis of the peak positions and integral line widths of the x-ray patterns showed that the assemblies formed by the two peptides have similar local packing and similar 30–40 Å-sizes or coherence lengths for their diffracting regions.

**Figure 1:**
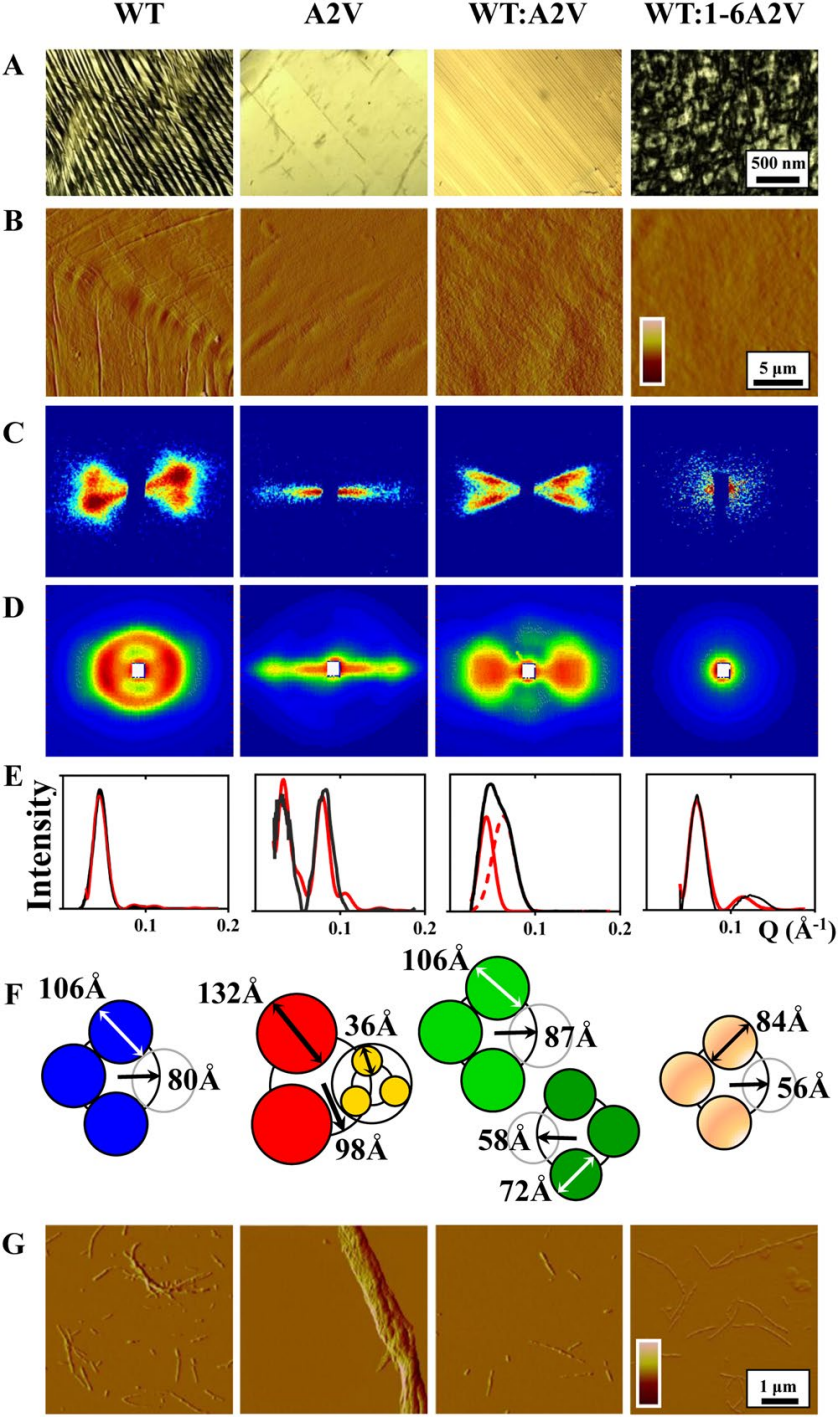
Biophysical characterization of the macromolecular assemblies formed by amyloidogenic peptides: WT; A2V; WT:A2V, a 1:1 molar mixture of the WT and A2V peptides; and WT:1-6A2V, a 4:1 molar mixture of the WT and hexapeptide 1-6A2V. (**A**) Polarized light microscopy. Scale bar: 500 nm. (**B**) AFM. Scale bar: 5 µm (**C**) Small-angle x-ray diffraction. (Data collected at ESRF, Grenoble, FR.) (**D**) Neutron diffraction. (Data collected at ILL, Grenoble, FR.). (**E**) Intensity of the ND data along the equator. Black line: observed scatter; solid and dashed red lines: calculated scatter (see Supplementary Information, Theory) for the size of structural unit and its assembly as modeled in (F). (**F**) The fibrils modeled by solid cylinders arranged as shown (see Supplementary Information, Theory). (**G**) AFM from the samples examined by x-ray and neutron diffraction after resuspension in ddH_2_O. Scale bar: 1 µm. In (**A**–**D**), the vertical direction corresponds to the direction of the aligning magnetic field used in sample preparation.

To prepare amyloid fibrils with higher degree of macroscopic axial orientation, concentrated solutions (10 mg/mL) of the peptides WT, A2V, WT:A2V, and WT:1-6A2V in quartz cells (Supplementary Information, Fig. SI2) were aligned while drying in the presence of a magnetic field. As previously described, molecules that possess diamagnetic anisotropy will orient in a constant magnetic field (B)^19^; for polypeptides, the peptide bonds and aromatic residues are the major sources of anisotropy: examples include collagen^19^, diphenylalanine tubes^20^ and Aβ peptides^21^. When the orienting energy is sufficiently greater than the disorienting thermal energy (*kT*) due to Brownian motion, a high degree of orientation will occur^19^, and stronger magnetic fields will induce better alignment. For our peptides, we confirmed by polarized light microscopy (Fig. 1A) and AFM (Fig. 1B) that a 7 T magnetic field was sufficient to induce alignment of WT and A2V. The dried, closely-packed fibrils formed by A2V were highly-aligned, whereas WT and the mixture WT:A2V formed two populations of fibrils, oriented ~30–40° to one another. Moreover, co-incubation of WT and 1-6A2V produced poorly- or non-oriented, seemingly non-fibrillar assemblies.

To assess the mesoscale organization (i.e. between the atomic and macrolevel dimensions) of the assemblies that had been visualized above, we humidified the samples by equilibration against an atmosphere of 100% D_2_O (14 days at room temperature) and then examined them using x-ray (XRD; Fig. 1C) and neutron (ND; Fig. 1D) scattering (Sample Set I). ND measurements were repeated on independently prepared samples (Sample Set II) and gave similar results (Supplementary Information, Fig. SI3). The diffraction results were consistent with the qualitative observations about fibril orientation made by polarized light microscopy, and gave quantitative information about the mesoscale structure of the aggregates.

Both ND and XRD showed that the four kinds of samples differed in the shape and angular distribution of their diffracted intensities.

For A2V, these techniques revealed relatively sharp, highly-oriented scattering consisting of two strong intensity maxima extending from the center of the pattern along the equator, which is consistent with well-formed fibers that are predominantly oriented along the magnetic field (Fig. 1C and D, second column). For WT, by contrast, the diffraction patterns (Fig. 1C and D, first column) were more complex in appearance, in that the predominant intensity maximum was more arced than for A2V, indicating less well-oriented fibers. Weaker lateral interactions between fibrils could account for greater disorientation; and the doubled maximum indicates a double-orientation of fibers (31° tilt) which could arise from twisting, completely absent in A2V. For WT:A2V (the 1:1 molar ratio mixture of the two peptides), both XRD and ND patterns (Fig. 1C and D, third column) showed broad, butterfly-like crisscross scatter (27° tilt). Finally, for WT:1-6A2V (the 1:4 molar ratio mixture of WT and 1-6A2V), only very weak XRD and ND scattering was detected (Fig. 1C and D, fourth column), and this was mostly concentrated adjacent to the beam stop, and had low or no directionality, indicating that the scattering objects were mostly randomly distributed. Thus, the amount of highly-organized, well-ordered (or crystalline) material varied considerably among the four types of samples. The good agreement between ND and XRD patterns (see also below), despite the ∼100 fold size difference in their beam cross-section (∼1 cm^2^ vs. <1 mm^2^, respectively) suggests that the observed features are widespread over the entire sample. For both ND and XRD, the absence of meridional reflections in the small-angle region indicated that the scattering objects —i.e., the structural units constituting the fibers— resemble elongated solid cylinders. No appreciable periodicity could be detected along the axial direction, at least on a length-scale of a few tens of Å. Should the structural unit itself be distorted around its axis, any axial periodicity would be obscured by spatial averaging. Therefore, in the following, the units were modelled as simple cylinders, and some of the folded polypeptide assumed to be arranged at some distance from the fiber axis, as described in the Methods section and in the literature quoted therein. More refined models can be found in the literature, offering details of the molecular and even of the single-aminoacid arrangement within the aggregates for different amyloidogenic peptides, which are based on molecular simulation owing to the lack of well developed crystals^22^, and deducing from NMR results the local contacts and proximities^23, 24^. The present experiments indicate the presence of differences in the mesoscale spatial arrangement of organized units of two variants of an extended Aβ fragment (1–28), including the N-terminal portion, and of their mixture. Differences in the interaggregate scale organization could hierarchically reflect differences in packing, which may be relevant to the onset and progression of the disease connected to the full-length Aβ peptide. Extending our analysis to use highly refined atomic models, developed for other fragments or other amyloidogenic peptides and for different purposes is not useful here, as it would require introducing a number of additional assumptions and specific parameters at much higher resolution than can be determined with confidence from our current data.

**Figure 2:**
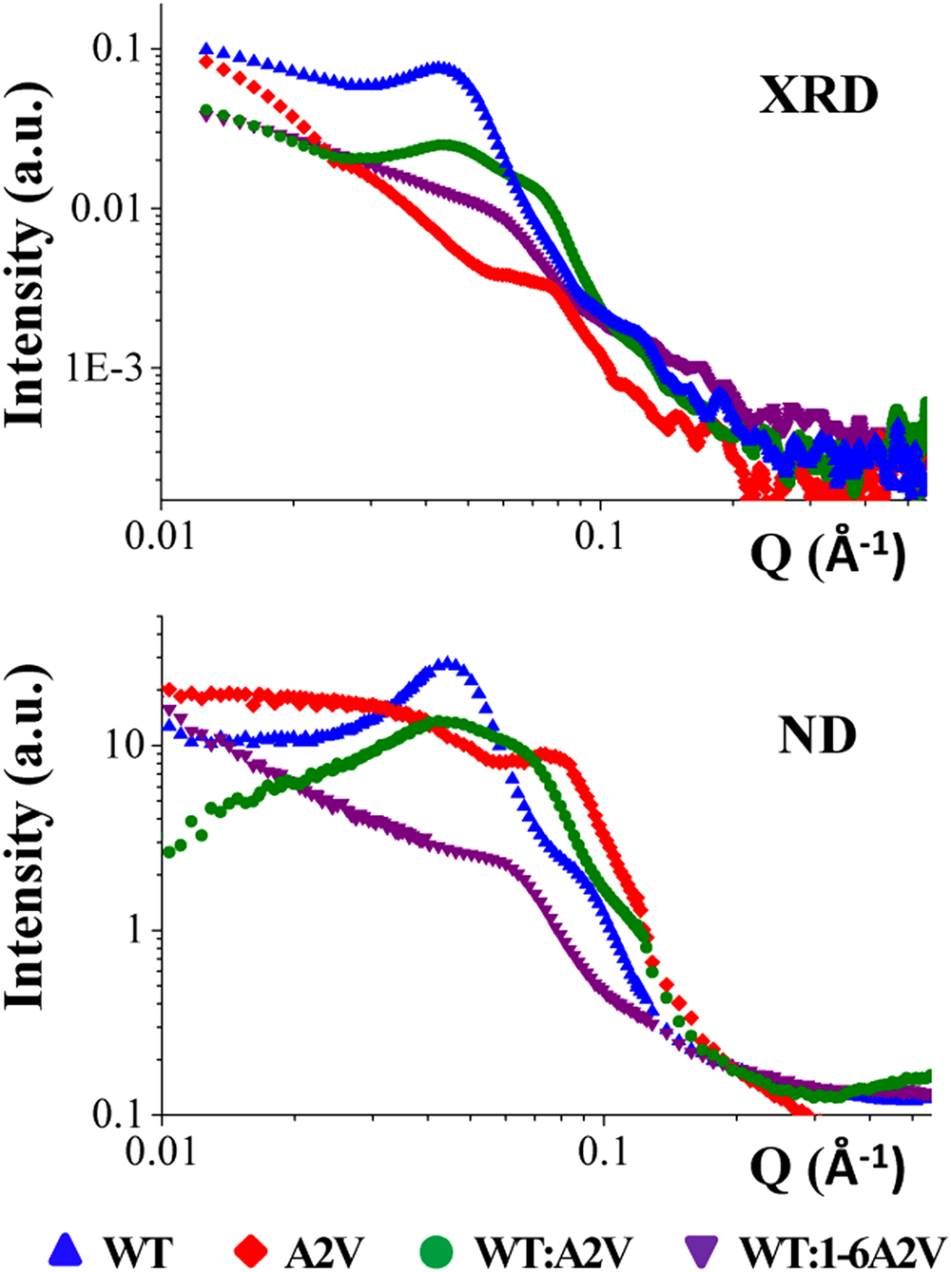
Comparison of equatorial x-ray (XRD) and neutron diffraction (ND) scatter from the WT, A2V, WT:A2V, WT:1-6A2V samples.

For Sample Set I, the ND and XRD scattering profiles along the equatorial (horizontal) direction (which is perpendicular to the fiber axis) showed close agreement between the two techniques (Fig. 2). The diffraction patterns for WT showed the main equatorial peak at Q = 0.045 Å^−1^ (corresponding to ~140 Å in real space), and a minor peak at Q = 0.09 Å^−1^. For A2V a broad intensity maximum extended over a range of a few-hundreds-Å and a less intense but sharper intensity maximum was at Q~0.08 Å^−1^ (corresponding to ~80 Å). The patterns from the mixture WT:A2V exhibited two peaks, one of which was similar in position to that of WT. For the mixture WT:1-6A2V, the peak was very weak, and positioned similar to one of the peaks in WT:A2V. Assuming these peaks arise from distances relating to inter-particle interference, then the separation of the scattering objects is largest in WT, and smallest in A2V. For WT and A2V, we also observed a weak reflection at Q~0.63 Å^−1^ (corresponding to 10 Å), which is the expected intersheet distance for β-sheet arrays. In the two mixed samples, however, a weaker peak was also present but for distances <10 Å. The smaller intersheet packing would be accounted for by closer packing of apposed β-sheets owing to smaller side chains and/or to a difference in side chain interactions. Table 1 lists the spacings observed for the reflections for each sample.

**Table 1:** Parameters deduced from Neutron and x-ray Diffraction Patterns.

	ND			XRD		
	Peak position on equator (Q in Å^−1^)	Characteristic distance in real space (d in Å)	Crystallinity *p*	Peak position on equator (Q in Å^−1^)	Mutual orientation (°)	Peak position on meridian (Q in Å^−1^) and [d in Å]
**WT**	0.045; 0.09	140; 70	0.45	0.044	31°	1.312 [4.79]
**A2V**	0.013–0.04; 0.08; 0.63	480–160; 80; 10	0.14	0.075		1.312 [4.79]
**WT:A2V**	0.047	133	0.47	0.044; 0.071	27°	
**WT:1–6A2V**	0.06	105	0.09	0.058		

As indicated above, the amount of crystallinity among the samples varied. To quantitate the amount of crystallinity, we calculated the relative contribution of fibrils to the total scatter, which included fibrils, oligomers, and monomers (Supplementary Information and Table 1). The excess intensity due to the crystallized material, based on the assembly of axisymmetric structural units, was compared to the unstructured background. The fibril contribution to the scatter from the samples, expressed by the crystallinity parameter *p*, was largest in WT and WT:A2V, and smallest in A2V and WT:1-6A2V. The estimated low crystallinity of the A2V sample seems to be in contrast with the high extent of fiber formation and alignment otherwise observed. This could be connected to the scarce water penetration into the deposited A2V sample, giving rise to lower contrast. In the following we will point out further observations in support of this explanation.

Analysis of the equatorial scatter (Fig. 1E) also provided sufficient data to calculate the widths of the structural units, assumed cylindrical, and of their spatial arrangement (described in the Supplementary Information, Theory). The lateral hindrance of the axisymmetric units, which best fit the equatorial diffracted intensities, were not the same for the different types of samples. A pictorial sketch is shown in Fig. 1F. For WT, the data were most consistent with four units, each 106 Å in diameter, organized on a circle 160 Å in size. For A2V, by contrast, a composite structure could be determined, where three narrow units, each 36 Å in diameter, were constituted in a 132 Å-size cylindrical volume that in turn was organized with two others onto a circle of diameter 196 Å. The equatorial scatter from WT:A2V indicated a superposition of two arrangements, indicating the coexistence of different structures. Finally, sample WT:1-6A2V had a very low content of fibrils, the few present retaining some common features with WT. In general, the sizes deduced here for the diffracting structural units were consistent with previous studies on protofilament or fibrillar, multimeric assemblies by x-ray and neutron solution scattering from serum amyloid P^25^, x-ray fiber diffraction from APP fragments Aβ1-28^26^ and Aβ1-40^21^, and solid-state NMR data on Aβ1-40^27, 28^. As already pointed out, direct structural information on lengths shorter than mesoscale are beyond the accessible range with the experimental techniques used here. Nonetheless, the calculated numerical values and the sketches reported in Fig. 1F can be examined to unveil possible analogies and discrepancies underlying the observed packing of variants, refering hierarchically to the mesoscale. In particular, due to the better definition of A2V aggregates, a more detailed description was allowed, detecting the existence of a smaller-size structural unit as compared to WT, having a size compatible with  a hairpin folded Aβ1-28 fragment^22^. Molecular modeling of this fragment has shown that the secondary structure propensity of the constituent amino acids allows for a turn in the intermediate 14–15 position, joining two adjacent sequences each displaying pronounced β-strand propensity. Moreover, the A to V substitution in position 2 is found to generate an important change in the free energy landscape of the peptide^22^. Notably, the size of the smallest detected unit in WT is three times as large as the one in A2V −106 Å vs 36 Å. Detailed existing models^24, 29^ address the existence of some general packing features shared by all amyloidogenic peptides, independent of their individual configuration. Spatial repetition and assembling of subunits is a common structural property, not only on the molecular scale, in the formation of β-sheet and in protofibrillar association, but also in lateral association of axially organized subunits, which gives rise to quantized sizes of aggregates. In this context, we then speculate that axially organized subunits of the same size (36 Å) are formed in both variants, but show a different ability to establish interactions between subunits, with WT arranging in the form of triplet-ribbons, and A2V in the form of triplet-bunches, a more condensed structure. This arrangement with a bunched-type cross-section has been observed within the polymorphic morphology of amyloid fibers^30^. A triplet-bunch versus triplet-ribbon packing could also account for the apparent higher rigidity of A2V fibers, favouring a compact macroscopic deposition that is precluded from twisting, by contrast with WT oriented fibers.

At wide-angles, which corresponds to a length-scale of a few Angstrom, both WT and A2V (Sample Set I) gave a distinct sharp meridional reflection. Angular integration of the meridional intensity revealed a peak centered at Q = 1.312 Å^−1^, corresponding in real space to d~4.8 Å, although weaker for the WT sample, confirming that both variants form β-sheet structures along the H-bonding direction and are more ordered for A2V. The wide-angle diffraction pattern and the angular integrated intensity are shown in Fig. SI4 for the A2V sample. Interestingly, the mixtures WT:A2V and WT:1-6A2V did not show this meridional reflection, indicating for their assemblies a greater disorder in the H-bonding direction. This finding helps in discriminating that the mixed WT:A2V fibers, although constituted by coexisting populations with different mesoscale packing, are truly mixed on the molecular scale with actual structural interference between the two variants. It can be speculated that the starting seed of the two populations is determined by either variant. This structural observation on the mixed fibers agrees and reinforces a previous finding on mixed oligomers, that they are readily soluble upon dilution, for longer times with respect to both constituents^10^. The absence of wide-angle diffraction from the WT:1-6A2V sample parallels the almost total absence of fibers detected in the small-angle profiles.

H-D exchange has been used to probe the core structures for an assortment of amyloid fibrils and protofibrils, in relation to the kinetics of molecular recycling within an ensemble of forming fibrils^31^, and based on the fact that the velocity of H-D exchange of the backbone amide hydrogens of Aβ is strongly reduced when monomers are incorporated into fibrils^32, 33^. This “conventional use” of the technique was performed while fibers were put in disaggregating solvent (DMSO), in order to follow the amount of free monomers able to leave the aggregated structure at different times and conditions. Additionally, we followed the H-D exchange rate on formed deposited fibers, in order to assess the accessibility of the aggregated structure and the extent of involvement of amide groups in internal engagement.

**Figure 3:**
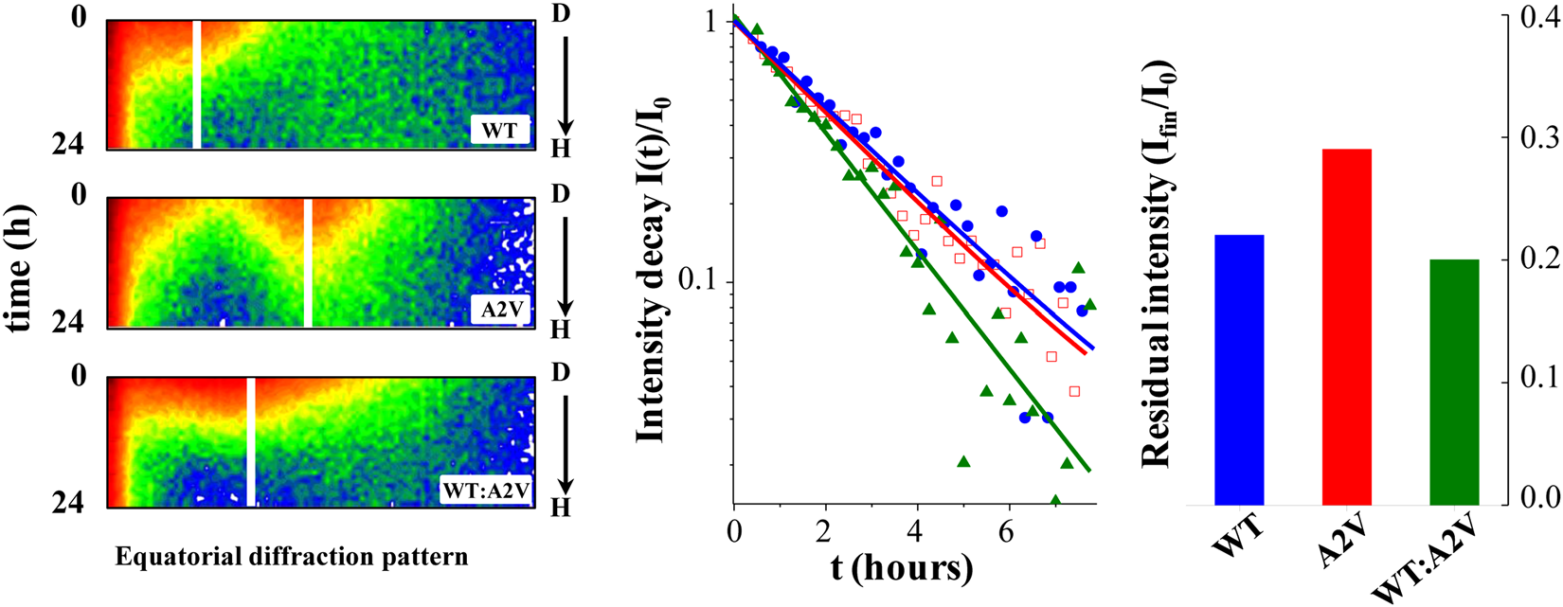
Analysis of neutron scattering changes due to H-D exchange in WT, A2V, and WT:A2V samples. (*Left*) Time-dependent change (D-H) in neutron scattering upon replacement of D_2_O with H_2_O in the tubular reservoir of the sample holder. (*Middle*) The decay in intensity of the predominant neutron scattering peak, *I(t)/I*_*o*_, during about eight hours, was fit by an exponential. The exchange times for WT, A2V, and WT:A2V, were 2.58 h, 1.90 h, and 2.45 h, respectively. (*Right*) Histogram showing the residual intensity of the major peak, calculated from the asymptote of the plateauing intensity after D_2_O → H_2_O exchange.

In the current study we measured the rates of H-D exchange to determine the propensity of the formed fibers to interact with solvent (Fig. 3). For WT, at time t = 0, after the system was equilibrated against 100% D_2_O, the intensity of neutron scattering from the predominant peak (Q = 0.045 Å^−1^) was high; however, upon substitution of D_2_O with H_2_O, the intensity started decreasing immediately and rapidly and, at the same time, the background intensity, due to the incoherent scatter by hydrogen incorporated into the sample, increased (Fig. SI6, *left panel*). As no shift in peak position was observed, this diffraction peak likely derives from amide hydrogens. These general features were also displayed for A2V (peak position at Q = 0.075 Å^−1^) and WT:A2V (peak position at Q = 0.047 Å^−1^). By contrast, we observed a difference in extent and kinetics of exchange, indicating that the organization within aggregates was not the same among the samples. The rates of H-D substitution for the different samples (Fig. 3, *middle panel*) were compared after normalizing the decay in intensity, I(t)/I_0_, and showed that the D-H exchange had similar kinetics in the WT and A2V assemblies, with characteristic decay times ~2.5 h. For the mixture of WT:A2V, however, exchange was faster, with a time of ~1.9 h indicating a weaker involvement of hydrogens in inter-aggregate H-bond formation, and greater exposure to the medium. After D-H exchange the residual intensity of the predominant peak which, as described above, reflects amide hydrogens was higher for the A2V sample (Fig. 3, *right panel*), indicating that a greater number of hydrogens are protected (or cryptic) within the amyloid fibrils, rather than exposed to the surrounding medium. This observation agrees with the ribbon versus bunches hypothesis.

Following the neutron diffraction experiments, the samples were resuspended in ddH_2_O (described in Methods) and re-examined by AFM to determine the size of the fibrils (Fig. 1G). The sample containing mixture WT:1-6A2V was easily and completely resuspended, more so than WT and mixture WT:A2V. By contrast, sample A2V could not be solubilized even after incubation for 3 days. The assemblies of A2V after resuspension were large macro-fibrillar complexes, while in the case of WT, both single and twisted macro-fibrils were observed. The mixture WT:A2V showed both twisted and single fibrils, most of which were short and/or broken. Finally, the mixture WT:1-6A2V showed single fibrils only. The estimated heights of the aggregates were 50–150 Å for WT and WT:A2V, ~350 Å for A2V, and 15–25 Å for WT:1–6A2V. The larger value for A2V is consistent with the sizes estimated from the low-angle ND and XRD (Fig. 1F). Again, these observations agree with the overall landscape presented so far.

## Conclusion

Astonishingly different structural features are observed in the aggregated structures of the examined Aβ1-28 fragments, differing by a single aminoacid (either alanine or valine) in the N-terminal position 2. These variants correspond to clearly different neuropathological and biochemical profiles connected to the whole Aβ peptide. Differences are found in the mesoscale arrangement, involving the interaction between elementary axial units, resulting in fibers of different compactness and solvent accessibility. Close structural interference is found when the two variants are mixed, which has a distinctive clinical correlate.

Taken together, these data suggest that the substitution of alanine by valine at position 2 of the amyloidogenic peptide affect the lateral interactions between fibrils, promoting a more compact, aligned and uniform structure. Further, the structure and characteristics of the mixture WT:1-6A2V indicated that the presence of the short N-terminal domain of mutated Aβ1-6A2V could disrupt or prevent inter-fibrillar interactions in the WT.

According to the models proposed based on NMR^27, 28^, the N-terminal residues are always located at the outside of the fibril. Thus, alanine-alanine interaction in WT and valine-valine interactions in A2V should be involved in inter-fibrillar interactions and dimerization. The higher degree of orientation in the latter suggests tighter packing or less sterically-hindered interference between laterally-adjoining protofilaments or fibrils. An interaction of alanine2 in the WT with valine2 in the variant peptide may disrupt the fibril interactions owing to a mismatch of the sidechains and steric hindrance that disrupts the hydrogen-bonding and inter-sheet interactions, and thereby prevent fibrillogenesis, which is one of the pathological features of AD.

In summary, our x-ray and neutron diffraction experiments using magnetically aligned samples, complemented by polarized light microscopy, AFM, and modeling, provide a rational basis for the paradoxical effects of A2V mutation in humans, explaining its aggressiveness in homozygous carriers and its prophylactic effect in heterozygous carriers. These findings should provide a basis for further development of A2V-modified Aβ peptides and peptido-mimetic molecules for therapeutic intervention in AD.

## Methods

### Sample preparation and wide-angle x-ray diffraction on lyophilized samples

The Aβ peptides were synthesized in-house using FMOC chemistry and purified by RP-HPLC (see methods and sequences below) purification as already described^34^. Neo Bio Science (Cambridge, Massachusetts) synthesized the same sequences to corroborate the reproducibility of the synthesis. Peptide purity was assessed by LC-MS and was always >95%.

The lyophilized peptides were gently packed into thin-walled glass capillaries of 0.7 mm (outer diameter) (Charles A. Supper, Inc., South Natick, MA). All glass capillaries were siliconized with Sigmacote^®^ (Sigma-Aldrich, St. Louis, MO) according to the method described previously^35^.

Measurements of x-ray diffraction patterns from the lyophilized samples were performed at room temperature using the Oxford diffraction Xcalibur PX Ultra system (Oxford Diffraction Ltd., 130 A Baker Avenue, Concord, MA 01742, USA)^36^. The CuKα X-ray beam was focused to 0.3 mm × 0.3 mm (full-width at half-maximum width at detector position). Spectra were collected with exposure time of 150 seconds on a two-dimensional Onyx CCD detector (Oxford Diffraction Inc., Concord, MA) with a sample-detector distance of 85 mm, covering the range for Bragg spacings d = 1.8–54 Å. After angular averaging, the background was fit by a polynomial curve to the intensity minima between the peaks, and subtracted. The subtracted spectra were then fit by multiple Gaussian peaks to measure the spacings, integral intensities, and integral widths.

### Peptide synthesis and alignment of samples for neutron and synchrotron x-ray diffraction

Aβ synthetic peptides homologous to residues 1–28 with or without the A-to-V substitutions at position 2 (Aβ1-28WT (WT) and Aβ1-28A2V (A2V), respectively) and Aβ synthetic peptide homologous to residues 1-6 with the A-to-V substitution (Aβ1-6A2V, (1-6A2V)) were synthesized by solid-phase peptide synthesis on an automated synthesizer (Applied Biosystems 433 A) using Fmoc-protected L-amino acid derivatives, NOVASYN-TGA resin on a 0.1 mM scale, and purified by reverse-phase HPLC using a Vydac Protein and Peptide C18 column (Grace Corporation, Passirana di Rho, Milano, IT). The purity and identity of peptides were determined by HPLC and MALDI-TOF analysis (model Reflex III, Bruker). Peptide purity was always above 95%.

The sequences of the peptides were:

$$\begin{array}{c}A{\rm{\beta }}1 \mbox{-} 28{\rm{WT}}({\rm{WT}}):{\rm{DAEFRHDSGYEVHHQKLVFFAEDVGSNK}}\\ \begin{array}{l}A{\rm{\beta }}1 \mbox{-} 28{\rm{A2V}}({\rm{A2V}}):{\rm{DVEFRHDSGYEVHHQKLVFFAEDVGSNK}}\\ A{\rm{\beta }}1 \mbox{-} {\rm{6A2V}}({\rm{1}} \mbox{-} {\rm{6A2V}}):{\rm{DVEFRH}}\end{array}\end{array}$$

The lyophilized WT and A2V peptides and their 1:1 molar ratio mixture (WT:A2V) were dissolved in ddH_2_O at 10 mg/mL and dehydrated at ambient temperature for four days on a quartz support in specially designed siliconized (Sigmacote^®^) glass cylindrical cells in the presence of a 7 T static magnetic field (Fig. SI2). This procedure led to the formation of fibrils and their alignment along the H-bonding direction between β-strands during fiber formation^19–21^. The N-terminus 1-6A2V peptide was co-dissolved with WT in ddH_2_O at 4:1 molar ratio and then submitted to the same procedure.

To assess orientation of the amyloid fibrils prior to x-ray and neutron diffraction experiments, birefringence of the thin, dried pellets was monitored by polarized light microscopy^21^.

The sample holders were then sealed at their tops with quartz windows (Fig. SI2B), 100% D_2_O was introduced into the tubular reservoirs to re-humidify the samples, and the samples were equilibrated by storage at room temperature for about 14 days prior to and during diffraction measurements.

This procedure was followed for preparing independently two sets of samples: Set I was structurally characterized using neutron diffraction and x-ray diffraction; Set II was characterized using neutron diffraction for structure determination and for D-H hydration kinetics. The different measurements on each sample set were carried out within a few hours of each other. Further, high reproducibility was observed between the two sample sets subjected to the same measurements.

Small-angle neutron diffraction (ND) experiments were performed at D22 (Set I), and D11 (Set II) and wide-angle neutron diffraction was performed on D16 (Set II) at the Institut Laue-Langevin (ILL, Grenoble, France). Full descriptions of the instruments can be found on the ILL website (https://www.ill.eu/instruments-support/instruments-groups/).

On the D11 and D22 beamlines, with wavelength of 4.75 Å and 4.5 Å, respectively, three sample-to-detector distances were selected (1.2 m, 6 m and 16 m for D11; 1.1 m, 5.6 m and 17.6 m for D22) in order to access a wide range of momentum transfer *Q*, being *Q* = *2π(1/d)* related to the Bragg spacing of the structure, *d*. On D16, with wavelength of 4.76 Å and sample-to-detector distance 0.95 m, the corresponding *Q*-range was 0.01–2.5 Å^−1^. The neutron diffraction patterns were visualized and analyzed with the ILL in-house developed program LAMP. Intensity distributions were derived by integrating over a sector of 10° opening, and were plotted along the horizontal and fiber (vertical) directions. The intensity was then radially integrated within the 10° sectors and the resulting scattering curves corresponding to the three sample-detector distances were merged.

To perform D-H exchange experiments at the D16 beamline, D_2_O was replaced with H_2_O in the tubular reservoir of the sample cell, which was then wax-sealed. Data were recorded during 30 hours, each point integrating over 15 min measurement time. The kinetics of D-H isotope replacement was estimated by recording the time evolution of the diffracted intensity in a *Q*-range corresponding to the predominant diffraction peak.

Synchrotron x-ray diffraction experiments were performed on ID02 beamline at the ESRF (Grenoble, France). The direct beam size was 0.3 × 0.8 mm^2^, the beam wavelength was 1 Å and the investigated Q-range was 0.002–0.5 Å^−1^ in the Small-Angle (SAXS) configuration and Q = 0.5–3.5 Å^−1^ in the Wide-Angle (WAXS) configuration. The two configurations allowed us characterize structural parameters on different length scales from interparticle to intraparticle distances. For each measurement, ten frames were taken with short exposure time (0.03 s, 1 s dead-time) to avoid or test for radiation damage. Contribution from the empty cell was measured and subtracted from the intensity diffracted by each sample in both configurations. Collected images were processed and corrected with the ID02 software SAXS utilities. Samples (Set I) were the same as used for the ND measurements on D22, and in the same siliconized glass cylindrical containers with quartz windows. Just before measurements on each sample, the upper quartz window was removed, and ddH_2_O water (3 μL) was locally added to the dry depositions, allowing for observable scattered x-ray intensity.

### Analysis of diffraction data

First, the background curves, evaluated by fitting polynomial functions to the intensity minima, were subtracted from the total intensity (Fig. 1E). Diffraction data were analyzed according to existing models suitable for aligned fibers with a regular spatial disposition that replicates the cylindrical shapes of the elementary structural units^37^. The complex structure of the subunits and their arrangements (Fig. 1F) were derived as a best fit by minimizing the *R*-factor between the intensity observed for the sample and intensity calculated for different models. The neutron scattering length density and electron density $\rho (\overrightarrow{r})$ distribution in three-dimensional coordinates, their autocorrelation function, and the corresponding calculated intensity distribution were expressed in cylindrical coordinates, and cylindrical spatial averaging was performed for the diffracted intensity

1 $${\langle I(R,Z=0)\rangle }_{cal}=[{f}^{2}(r,R,Z=0){{\rm{\Phi }}}^{2}({n}_{0},{r}_{0},R)]{\tilde{{\rm{\Phi }}}}^{2}({n}_{1},{r}_{1},R)$$

which accounts for the shape of the fibrils (of radius *r*), their spatial disposition in a first circular lattice (of radius *r*_0_ with *n*_0_ elements), and the further arrangement in cylindrical superstructure (of radius *r*_1_ with *n*_1_ elements). *R* is the reciprocal coordinate *R* = *Q*/2π. We considered different models, corresponding to different numbers of fibrils (lattice points *n*_0_ and *n*_1_)^38, 39^ composing the circular lattice of the two-steps cylindrical arrangement. Polydispersity in cylinder radius was allowed, with Gaussian distribution around a mean value^37^ The calculated intensity distribution from the model was then compared with the experimental intensity distribution (after background subtraction and normalization) by means of a deviation *R*-factor

2 $$R={\int }_{{R}_{1}}^{{R}_{2}}[{I}_{obs}(R)-{\langle I(R,Z=0)\rangle }_{cal}]dR/{\int }_{{R}_{1}}^{{R}_{2}}[{I}_{obs}(R)]dR$$

that was minimized to select the best-fit values of *r*, *r*_0_, *n*_0_, *n*_1_ and *n*_1_ A crystallinity parameter was also evaluated, defined as

3 $$p={\int }_{{R}_{1}}^{{R}_{2}}[I(R)-B(R)]\frac{dR}{{\int }_{{R}_{1}}^{{R}_{2}}I(R)dR}$$

which assessed the excess intensity due to the crystallized material as compared to the unstructured background.

### Atomic Force Microscopy

Atomic force microscopy (AFM) was used to both assess the alignment of fibrillar aggregates of Aβ after ND experiments (Fig. 1B) and to assess the morphology of aggregates after re-suspension (Fig. 1G). AFM was carried out on a Multimode AFM with a Nanoscope V system operating in tapping mode, using standard phosphorus-doped silicon probes (Veeco/Digital Instruments, Mannheim, Germany) with a scan rate in the 0.5–1.2 Hz range. Freshly cleaved muscovite mica discs (Veeco/Digital Instruments) were used for deposition of peptide samples. To confirm the extent of alignment and to provide more detailed information on the orientation of fibrillar aggregates, a portion of film was detached from each of the samples used for ND experiments and fixed on the surface of mica with a drop (2 µL) of ddH_2_O. In addition, to assess the morphology of the fibrillar aggregates, a piece of the dried film was taken from each of the samples, weighted, and re-suspended in ddH_2_O to an estimated final Aβ concentration of ~250 µM. After 8 hours of incubation, samples were further diluted to 5 µM. A 50 µL-aliquot of the resulting suspension was deposited onto mica and incubated for 2 min at room temperature, washed with 10 mL of water to remove the unbound sample, and dried under a gentle stream of nitrogen. The AFM images were analyzed by Scanning Probe Image Processor (SPIP-version-5.1.6).

## Electronic supplementary material

Supplementary Information

## References

[1] Hardy J, Selkoe DJ (2002). The amyloid hypothesis of Alzheimer’s disease: progress and problems on the road to therapeutics. Science.

[2] Fändrich M, Schmidt M, Grigorieff N (2011). Recent progress in understanding Alzheimer’s β-amyloid structures. Trends Biochem. Sci.

[3] Greenwald J, Riek R (2010). Biology of amyloid: structure, function, and regulation. Structure.

[4] Broersen K, Rousseau F, Schymkowitz J (2010). The culprit behind amyloid β peptide related neurotoxicity in Alzheimer’s disease: oligomer size or conformation. Alzheimer’s Res. Ther..

[5] Selkoe DJ, Hardy J (2016). The amyloid hypothesis of Alzheimer’s disease at 25 years. EMBO Mol. Med..

[6] Kayed R (2003). Common structure of soluble amyloid oligomers implies common mechanism of pathogenesis. Science.

[7] Walsh DM (2002). Naturally secreted oligomers of amyloid β protein potently inhibit hippocampal long-term potentiation *in vivo*. Nature.

[8] Amtul Z (2016). Why therapies for Alzheimer’s disease do not work: Do we have consensus over the path to follow?. Ageing Res. Rev..

[9] Schneider LS (2014). Clinical trials and late-stage drug development for Alzheimer’s disease: an appraisal from 1984 to 2014. J. Intern. Med..

[10] Di Fede G (2009). A recessive mutation in the APP gene with dominant-negative effect on amyloidogenesis. Science.

[11] Di Fede G (2016). Tackling amyloidogenesis in Alzheimer’s disease with A2V variants of Amyloid-β. Sci. Rep..

[12] Messa M (2014). The peculiar role of the A2V mutation in amyloid-β (Aβ) 1-42 molecular assembly. J. Biol. Chem..

[13] Di Fede G (2012). Good gene, bad gene: new APP variant may be both. Prog. Neurobiol..

[14] Diomede L (2016). The new β amyloid-derived peptide Aβ1-6A2V-TAT(D) prevents Aβ oligomer formation and protects transgenic C. elegans from Aβ toxicity. Neurobiol. Dis..

[15] Cimini S (2016). The cell-permeable Aβ1-6A2VTAT(D) peptide reverts synaptopathy induced by Aβ1-42wt. Neurobiol. Dis..

[16] Nyström S (2012). Multiple Substitutions of Methionine 129 in Human Prion Protein Reveal Its Importance in the Amyloid Fibrillation Pathway. J. Mol. Biol..

[17] Palmer MS, Dryden AJ, Hughes JT, Collinge J (1991). Homozygous prion protein genotype predisposes to sporadic Creutzfeldt-Jakob disease. Nature.

[18] Qiang W, Yau WM, Lu JX, Collinge J, Tycko R (2017). Structural variation in amyloid-β fibrils from Alzheimer’s disease clinical subtypes. Nature.

[19] Torbet J, Ronziere MC (1984). Magnetic alignment of collagen during self-assembly. Biochem. J..

[20] Hill ARJ (2007). Alignment of aromatic peptide tubes in strong magnetic fields. Adv. Mater..

[21] Malinchik SB, Inouye H, Szumowski KE, Kirschner DA (1998). Structural analysis of Alzheimer’s β(1-40) amyloid: Protofilament assembly of tubular fibrils. Biophys. J..

[22] Nguyen PH, Tarus B, Derreumaux P (2014). Familial Alzheimer A2V Mutation Reduces the Intrinsic Disorder and Completely Changes the Free Energy Landscape of the Aβ1−28 Monomer. J. Phys. Chem. B..

[23] Petkova AT (2002). A structural model for Alzheimer’s betaamyloid fibrils based on experimental constraints from solid state NMR. PNAS.

[24] Fitzpatrick AWP (2013). Atomic structure and hierarchical assembly of a cross-β amyloid fibril. PNAS.

[25] Ashton AW, Boehm MK, Gallimore JR, Pepys MB, Perkins SJ (1997). Pentameric and decameric structures in solution of serum amyloid P component by X-ray and neutron scattering and molecular modelling analyses. J. Mol. Biol..

[26] Inouye H, Fraser PE, Kirschner DA (1993). Structure of β-crystallite assemblies formed by Alzheimer β-amyloid protein analogues: analysis by x-ray diffraction. Biophys. J..

[27] Petkova AT, Yau WM, Tycko R (2006). Experimental constraints on quaternary structure in Alzheimer’s β-amyloid fibrils. Biochemistry.

[28] Lu JX (2013). Molecular structure of β-amyloid fibrils in Alzheimer’s disease brain tissue. Cell.

[29] Langkilde AE, Morris LK, Serpell LC, Svergun DI, Vestergaard B (2015). The architecture of amyloid-like peptide fibrils revealed by X-ray scattering, diffraction and electron microscopy. Acta Cryst..

[30] Meinhardt J, Sachse C, Hortschansky P, Grigorieff N, Fändrich M (2009). Aβ(1–40) Fibril Polymorphism Implies Diverse Interaction Patterns in Amyloid Fibrils. J. Mol. Biol..

[31] Carulla N, Zhou M, Giralt E, Robinson CV, Dobson CM (2010). Structure and intermolecular dynamics of aggregates populated during amyloid fibril formation studied by hydrogen/deuterium exchange. Accounts Chem. Res..

[32] Kheterpal I, Zhou S, Cook KD, Wetzel R (2000). Aβ amyloid fibrils possess a core structure highly resistant to hydrogen exchange. Proc. Natl. Acad. Sci. USA.

[33] Whittemore NA (2005). Hydrogen-deuterium (H/D) exchange mapping of Aβ1-40 amyloid fibril secondary structure using nuclear magnetic resonance spectroscopy. Biochemistry.

[34] Manzoni C (2009). Overcoming synthetic Abeta peptide aging: a new approach to an age-old problem. Amyloid.

[35] Inouye, H., Sharma, D., Kirschner, D. A. X-ray diffraction for characterizing structure in protein aggregates. In: *Misbehaving Proteins*., Springer, Murphy, R., and Tsai, A. (Eds.) 353p (2006).

[36] Sharma D, Shinchuk LM, Inouye H, Wetzel R, Kirschner DA (2005). Polyglutamine homopolymers having 8–45 residues form slablike beta-crystallite assemblies. Proteins: Struct., Funct., Bioinf..

[37] Inouye H (2014). Multiscale deconstruction of molecular architecture in corn stover. Scientific Reports.

[38] Inouye H, Worthington CR (1983). X-ray observations on a collagen fibril lattice structure in peripheral nerve. Int. J. Biol. Macromol..

[39] Vainshtein, B. K. *Diffraction of X-Rays by Chain Molecules*., Elsevier, London. 414 p (1966).

