# Supplementary material for: The A2V mutation as a new tool for hindering Aβ aggregation: A neutron and x-ray diffraction study
Source: Sci Rep. 2017 Jul 14;7:5510. doi: 10.1038/s41598-017-05582-9 (PMC5511251; doi:10.1038/s41598-017-05582-9)
Supplement: Supplementary file 1 — Supplementary Information [file 41598_2017_5582_MOESM1_ESM.pdf]

Supplementary Information

The A2V mutation as a new tool for hindering Aβ aggregation:A neutron and x-ray diffraction study

Laura Cantu',[1] Laura Colombo,[2] Tatiana Stoilova,[2] Bruno Demé,[3] Hideyo Inouye,[4] Rachel Booth,[5] Valeria Rondelli,[1] Giuseppe Di Fede,[6] Fabrizio Tagliavini,[6] Elena Del Favero,[1] Daniel A. Kirschner,[5] and Mario Salmona.[2]*

[1]Department of Medical Biotechnology and Translational Medicine, University of Milan, LITA, Segrate, 20090 Milano, Italy. [2]Department of Molecular Biochemistry and Pharmacology, IRCCS Istituto di Ricerche Farmacologiche "Mario Negri", Milano, 20156, Italy. [3]Institut Laue-Langevin, Grenoble Cedex 9, 38042, France. [4]Department of Electrical and Computer Engineering, Northeastern University College of Engineering, Boston, MA 02115, USA. [5]Biology Department, Boston College, Chestnut Hill, MA 02467-3811, USA. [6]Neurology V and Neuropathology Unit, IRCCS Foundation "Carlo Besta" Neurological Institute (INCB), Milano, 20133, Italy.

*To whom correspondence addressed: mario.salmona@marionegri.it

Contents

Supplementary Theory: Pages S2-S4

Supplementary Reference: Pages S5

Supplementary Figures: Pages S6-S9

Theory

Circular lattice

The diffraction data were analyzed according to existing models suitable for aligned fibers with a regular spatial disposition replicating the cylindrical shape of the elementary constituents. The neutron scattering length density and electron density $\rho(\vec{r})$ distribution in three-dimensional coordinates, its auto correlation function, and intensity distribution as a function of reciprocal coordinate $\vec{R}$ may be given by

$$\rho(\vec{r}) = \sum_{j=1}^{N} \rho_j(\vec{r}) * \delta(\vec{r} - \vec{r}_j)$$

$$\tilde{\rho}^2(\vec{r}) = \sum_{j=1}^{N}\sum_{k=1}^{N} \rho_j(\vec{r}) * \rho_k(-\vec{r}) * \delta\left[\vec{r} - \left(\vec{r}_j\text{-}\vec{r}_k\right)\right] \qquad (1)$$

$$I(\vec{R}) = \sum_{j=1}^{\mathrm{N}}\sum_{k=1}^{\mathrm{N}} f_j(\vec{R}) f_k^*(\vec{R}) \mathrm{esp}i2\pi\delta\left(\vec{r}_j - \vec{r}_k\right)\vec{R}$$

When the real and reciprocal vectors are expressed in cylindrical coordinates (*r*, ϕ, *z)* and (*R*, Φ, *Z)*, cylindrical averaging of the exponential term gives

$$< \mathrm{exp}i2\pi\vec{r}_{jk}\vec{R} >_{\Phi} = \left\{\frac{1}{2\pi}\int_{\Phi=0}^{2\pi} \mathrm{exp}i\,2\pi\left[r_{jk}R\cos\left(\emptyset_{jk} - \Phi\right)\right]d\Phi\right\} expi2\pi z_{jk}Z$$

$$= \left(\mathrm{exp}i2\pi z_{jk}Z\right)J_0\left(2\pi r_{jk}R\right) \qquad (2)$$

where J_0 is a zero-order Bessel function. Then, the cylindrically averaged intensity is expressed as

$$< I(R,Z) >= \left[\sum_j\sum_k f_j(R,Z) f_k(R,Z) J_0\left(2\pi r_{jk}R\right)\right] expi2\pi z_{jk}Z \qquad (3)$$

This is the cylindrical case of the Debye formula.

Some examples are given below for the cylindrically averaged intensity of the restricted lattice according to the equation $\Phi^2(R) = \sum_{j=1}\sum_{k=1} J_0\left(2\pi r_{jk}R\right)$ where $\vec{r}_j = \left|\vec{r}_j - \vec{r}_k\right|$ in cylindrical coordinates for

fibril positions *m,n*.[1] These structure factors have been tabulated in Vainshtein.[2] For a solid cylinder with radius *r*and length *L*, the $f_j(R, Z)$ can be written as

$$f_j(R,Z) = f(R,Z) = \frac{r_0 J_1\ (2\pi rR)}{R}\frac{\sin\pi LZ}{\pi Z} \qquad (4)$$

When the length of a solid cylinder is large, the structure factor can be restricted to *Z=0*. Then the cylindrically averaged intensity can be written as

$$< I(R,Z=0) >_{cal} = f^2(r,R,Z=0)\Phi^2(n,r_0,R) \qquad (5)$$

Here it is assumed that the cylinders are parallel, and the axial displacement is zero. The distance between the lattice points on a circle a is related to the radius of a circle r_0 by $a = 2r_0\sin(\pi / n)$. For different numbers of lattice points e.g., (6) two, (7) three, (8) four, (9) five, etc. the cylindrically-averaged intensities are:

$$\Phi^2(R) = \sum_{m=1}^{2}\sum_{m=1}^{2} J_0\ (2\pi r_{mn}\ R) = 2[1 + J_0(2\pi aR)] \qquad (6)$$

$$\Phi^2(R) = \sum_{m=1}^{3}\sum_{m=1}^{3} J_0\ (2\pi r_{mn}\ R) = 3[1 + 2J_0(2\pi aR)] \qquad (7)$$

$$\Phi^2(R) = \sum_{m=1}^{4}\sum_{m=1}^{4} J_0\ (2\pi r_{mn}\ R) = 4\left[1 + 2J_0(2\pi aR) + J_0\left(2\pi\sqrt{2}aR\right)\right] \qquad (8)$$

$$\Phi^2(R) = 5 + 8J_0\ (2\pi aR) + 8J_0\left(\pi\sqrt{2}aR\right) \qquad (9)$$

$$\Phi^2(R) = 5 + 10J_0(2\pi aR) + 10J_0(2\pi \bullet 1.618\ aR) \qquad (10)$$

$$\Phi^2(R) = 6 + 10J_0(2\pi \bullet 0.851\ aR) + 10J_0(2\pi aR) + 10J_0(2\pi \bullet 1.618\ aR) \qquad (11)$$

$$\Phi^2(R) = 6 + 12J_0(2\pi aR) + 12J_0\left(2\pi\sqrt{3}aR\right) + 6J_0(4\pi aR) \qquad (12)$$

$$\Phi^2(R) = 7 + 24J_0(2\pi aR) + 12J_0\left(2\pi\sqrt{3}aR\right) + 6J_0(4\pi aR) \qquad (13)$$

The relative deviation between the observed and calculated intensities after normalization is

$$R = \int_{R_1}^{R_2}[I_{obs}(R) - < I(R,Z=0) >_{cal}]\, dR / \int_{R_1}^{R_2}[I_{obs}(R)]dR \qquad (14)$$

The *R*-factor was calculated as a function of r, r_0, *n* and the smallest *R*-factor was searched in order to derive the optimum values for these parameters.

The composite assembly of multiple circular lattices is described by

$$< I(R,Z=0) >_{cal} = \left[f^2(r,R,Z=0)\Phi^2(n_0,r_0,R)\right]\tilde{\Phi}^2(n_1,r_1,R) \qquad (15)$$

where $f(r, R, Z = 0)$ is the Fourier transform at Z = 0 for a solid cylinder of radius r, Φ^2 (n_0, r_0, R), is the interference function of circular lattice of radius r_0 and the number of lattice points of n_0, $\vec{\Phi}^2$ (n_1, r_1, R) is the interference function of a larger circular lattice of radius r_1 and the number of lattice points of n_1. The relative deviation between the observed and calculated intensities is defined as

$$R = \int_{R_1}^{R_2}[I_{obs}(R) - < I(R, Z = 0) >_{cal}]\, dR / \int_{R_1}^{R_2}[I_{obs}(R)]\, dR \quad (16)$$

The R-factor was calculated as a function of r, r_0, r_1, n_0 and n_1 and the smallest R-factor was searched in order to derive the optimum values.

Low-angle scattering

The low-angle scattered intensity at equator was interpreted as arising from the Fourier transform of a solid cylinder. The intensity distribution of solid cylinders having different radii is

$$I(R) = \left\{\int_{r=0}^{\infty}\left[\frac{rJ_1(2\pi rR)}{R}\right]^2 \frac{1}{\sqrt{2\pi}\sigma_r} exp\left[-\frac{(r_0 - r)^2}{2\sigma_r^2}\right] dr\right\} \quad (17)$$

where R, Z are radial and axial components of cylindrical reciprocal coordinates, r is a radius of a solid cylinder, r_0 is the mean radius, and σ is standard deviation.[3] The reciprocal axial direction is parallel to the axial direction in real space. The fiber axis was chosen as the meridional direction. The equatorial streak normal to the axis was interpreted as arising from the solid cylinders (the structural units here) aligned along the principal fiber axis.

Crystallinity

The crystallinity p was defined as

$$p = \int_{R_1}^{R_2}[I(R) - B(R)]\, dR / \int_{R_1}^{R_2} I(R)\, dR \quad (18)$$

Where $I(R)$ is the observed equatorial intensity distribution as a function of reciprocal coordinate R, and $B(R)$ is the background intensity distribution approximated by polynomials. The intensity curves were integrated in the reciprocal range of R_1 and R_2.

References

(1) Inouye, H., Worthington, C. R. X-ray observations on a collagen fibril lattice structure in peripheral nerve. *Int. J. Biol. Macromol*. **5**, 199-203 (1983).

(2) Vainshtein, B. K. *Diffraction of X-Rays by Chain Molecules*. Elsevier, London. 414 pp (1966).

(3) Inouye, H., Zhang, Y, Yang, L., Venugopalan, N., Fischetti, R. F., Gleber, S. C., Vogt, S., Fowle, W., Makowski, B., Tucker, M., Ciesielski, P., Donohoe, B., Matthews, J., Himmel, M. E., Makowski, L. Multiscale deconstruction of molecular architecture in corn stover. *Scientific Reports*, **4**:3756 (2014).

Supplementary Figures

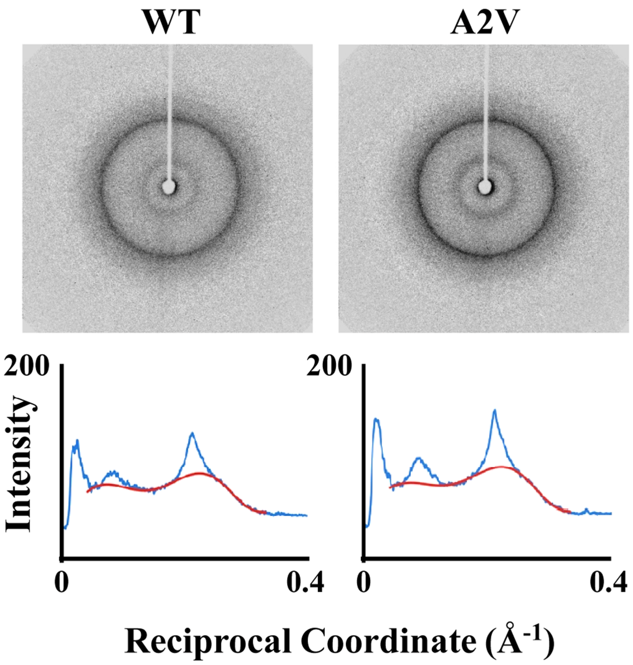

Figure SI.1. X-ray diffraction patterns (top) from the wild type (WT) and A2V sequences for the 28 amino acid-long fragment of Aβ1-40/42 i.e., WT and A2V, and radially-integrated intensities of the patterns. The peak positions corresponded to spacings of 11.52 Å and 4.66 Å for WT, and to 10.73 Å and 4.68 Å for A2V; and the integral linewidths (Gaussian fit) were 1/32.2 Å and 1/36 Å for WT, and 1/29.5 Å and 1/37.4 Å for A2V. The calculated coherence lengths (or sizes of the diffracting region, or crystallinity), were ~30 Å and ~40 Å. These x-ray diffraction patterns were obtained at room temperature using the Oxford diffraction Xcalibur PX Ultra system (Oxford Diffraction Ltd., 130A Baker Avenue, Concord, MA 01742, USA) in the laboratory of Dr. Andrew Bohm (Department of Biochemistry, Tufts University, Boston, MA).

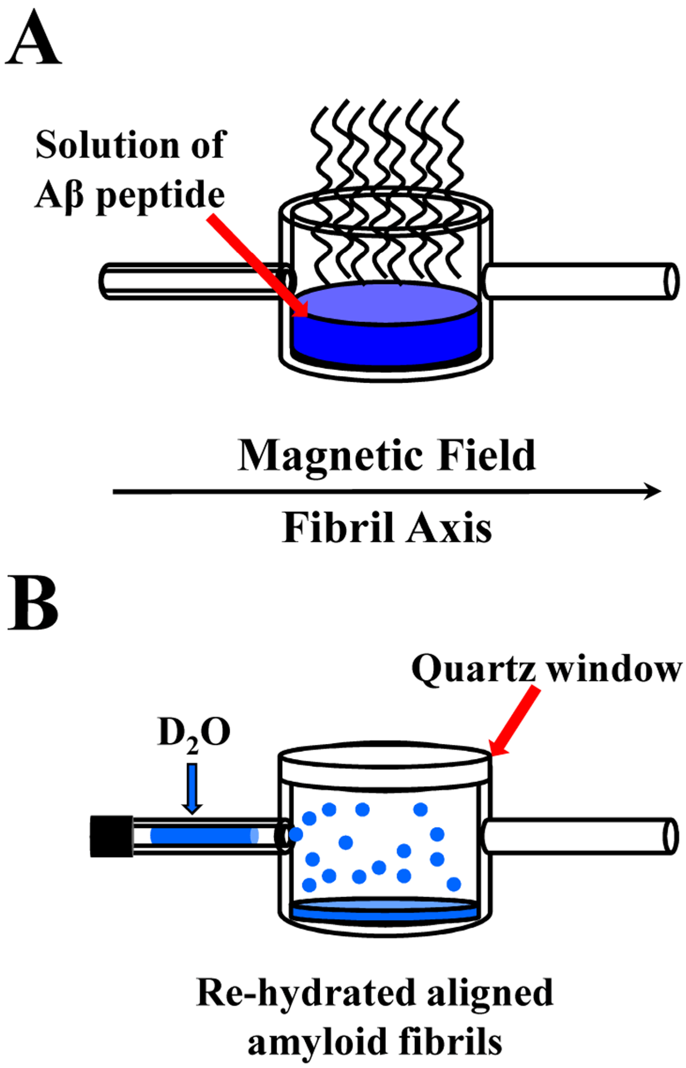

Figure SI.2. Schematics showing the glass chambers used for drying the peptide solutions. The tube to the left of the chamber was used as a solution reservoir for D_2O-H_2O exchanges. The upper, quartz window of the chamber, which was used to seal the chamber, was demountable for easy access in loading the chamber with solution, or allowing the water to evaporate during equilibrium and alignment in the external, 7 Tesla magnetic field, the direction of which is indicated by the horizontal arrow. When oriented, the fibers were aligned in the direction of the inter-β-strand H-bonding, i.e., along the long axis of the fibers. Dimensions of the chamber were 2 cm diameter by 1.5 cm height.

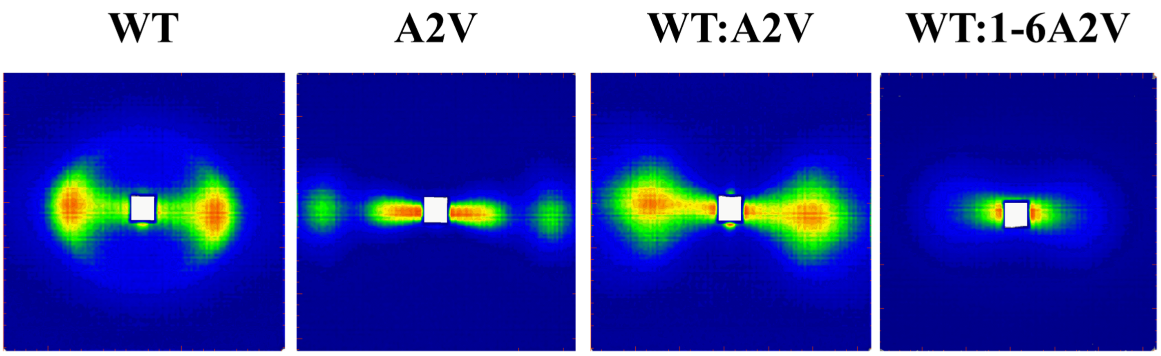

Figure SI.3. Neutron diffraction patterns from Sample Set II (WT, A2V, WT:A2V, WT:1-6A2V) recapitulated the features of the corresponding samples from Set I (Fig. 1D).

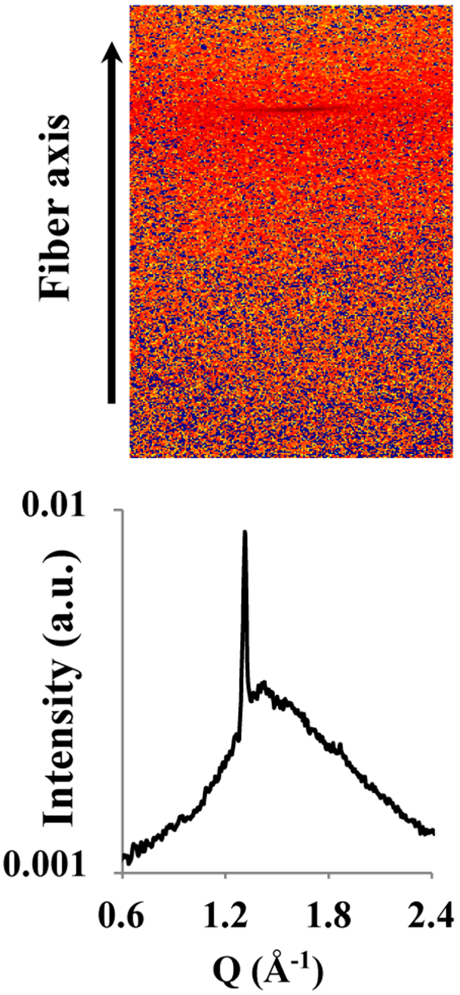

Figure SI.4. Meridional x-ray scatter from A2V sample. The raw intensity data (above), after subtraction of the scatter from the sample cell, shows a very sharp reflection that is predominantly on the meridian and has little arcing, indicating a high degree of orientation of the A2V assemblies. Angular integration of the intensity reveals this reflection is centered at a spacing of 4.8 Å.
